# The Physiologically Difficult Airway

**DOI:** 10.5811/westjem.2015.8.27467

**Published:** 2015-12-08

**Authors:** Jarrod M. Mosier, Raj Joshi, Cameron Hypes, Garrett Pacheco, Terence Valenzuela, John C. Sakles

**Affiliations:** * University of Arizona, Department of Emergency Medicine, Tucson, Arizona; † University of Arizona, Department of Medicine, Division of Pulmonary, Critical Care, Allergy and Sleep, Tucson, Arizona

## Abstract

Airway management in critically ill patients involves the identification and management of the potentially difficult airway in order to avoid untoward complications. This focus on difficult airway management has traditionally referred to identifying anatomic characteristics of the patient that make either visualizing the glottic opening or placement of the tracheal tube through the vocal cords difficult. This paper will describe the physiologically difficult airway, in which physiologic derangements of the patient increase the risk of cardiovascular collapse from airway management. The four physiologically difficult airways described include hypoxemia, hypotension, severe metabolic acidosis, and right ventricular failure. The emergency physician should account for these physiologic derangements with airway management in critically ill patients regardless of the predicted anatomic difficulty of the intubation.

## INTRODUCTION

The “difficult airway” has traditionally been used to describe intubations that have anatomic characteristics that make visualization of the vocal cords and placement of the tracheal tube challenging. Although scoring systems and prediction rules to identify the potentially difficult airway may be helpful, the performance of these prediction methods is only moderately successful. Additionally, the last decade has seen an incredible expansion of devices available to successfully ventilate, visualize the vocal cords, and place a tracheal tube leaving these prediction methods less useful.^1^ However, even with the expansive armamentarium available for emergent airway management, contextual factors such as operator experience, time pressures, and the patient’s underlying physiologic alterations still often result in difficulty with optimizing gas exchange, which is the primary goal of airway management.^2^

Critically ill patients represent the highest risk patients to intubate because of these contextual factors that increase the incidence of adverse events leading to dangerous hypoxemia, hemodynamic collapse and cardiac arrest.^3^–^11^ This baseline physiologic risk is exaggerated when intubations require more than one attempt,^12^–^15^ with difficult intubations being an independent predictor of death.^16^ As a result of the higher risk of these untoward events at intubation, first pass success has become the goal. Research in airway management has lead to advances that have greatly improved the management of the anatomically difficult airway, yet critically ill patients remain high-risk patients due to underlying pathophysiologic abnormalities. While the anatomically difficult airway is one in which obtaining a glottic view or passing an endotracheal tube is challenging, the physiologically difficult airway is one in which physiologic derangements place the patient at higher risk of cardiovascular collapse with intubation and conversion to positive pressure ventilation. These physiologic derangements should be accounted for in the intubation plan even if one does not predict anatomic difficulty with intubation. This paper will review four clinically important physiologically difficult airways that the emergency physician will encounter: hypoxemia, hypotension, severe metabolic acidosis, and right ventricular failure. Unfortunately, the physiologically difficult airway is not well described and there are very limited data available on management methods. In this paper we will provide physiologically and experience-based recommendations and, where available, evidence-based recommendations to decrease the risk of hemodynamic collapse when faced with one of these four high-risk airway management scenarios.

### Hypoxemia

Hypoxemic respiratory failure (Type I) in which there is failure to maintain adequate arterial oxygenation is a relatively common indication for intubation and invasive mechanical ventilation in the emergency department (ED). The mechanism of acute hypoxemic respiratory failure is most commonly due to any etiology that disrupts optimal alveolar-capillary gas exchange, such as pneumonia, acute respiratory distress syndrome (ARDS), and cardiogenic or non-cardiogenic pulmonary edema. In each of these instances a portion of the blood passing through the pulmonary circulation shunts past the remaining functional alveoli without the opportunity to participate in gas exchange. This hypoxemia is different than that which occurs with hypercapneic respiratory failure (Type II), which is due to decreased alveolar ventilation or an increase in dead space. Hypoxemia from Type II respiratory failure is relatively easily corrected with supplemental oxygen or an increase in minute ventilation. Hypoxemic respiratory failure patients are at high risk for rapid desaturation during intubation, which may result in hemodynamic instability, hypoxic brain injury, and potentially cardiopulmonary arrest.^7^,^17^–^19^ Identification of patients at risk for desaturation, such as the patient with limited reserve from acute hypoxemic respiratory failure or obesity, and utilization of all techniques available to prolong this time to desaturation, or safe apnea time, regardless of one’s assessment of the anatomic difficulty of the intubation is critical for the emergency physician.

Preoxygenation is an important step in every intubation with the goals of achieving the following: 1) maximal hemoglobin saturation and, 2) maximal partial pressure of arterial oxygen.^20^,^21^ The current standard method of preoxygenation includes the use of a non-rebreather (NRB) mask with tidal breathing for 3–5 minutes. This standard was extrapolated from studies in the operating room that used tight-fitting facemasks that prevented any air leak from the anesthesia circuit.^22^–^25^ Safe apnea time is prolonged with preoxygenation, but variable depending on factors that change the rate of oxygen consumption or functional residual capacity (FRC) such as critical illness and obesity.^26^,^27^ While a NRB provides an oxygen reservoir designed to breathe a higher fraction of inspired oxygen (FiO_2_), the rigid mask does not create an adequate seal and thus ambient air is entrained around the mask and decreases the effective FiO_2_ to much less than 100%. The higher the minute ventilation, the more this ambient air dilutes the FiO_2_ by admixing with the oxygen reservoir from the NRB. This relationship of FiO_2_ with underlying minute ventilation makes preoxygenation with a NRB less effective in critically ill patients. Noninvasive positive pressure ventilation (NIPPV) has been shown to improve oxygenation beyond usual preoxygenation methods, particularly in patients with obesity and shunt physiology.^28^,^29^ NIPPV increases mean airway pressure with the benefit of alveolar recruitment, temporarily decreasing shunt fraction and improving oxygenation.^28^,^30^–^33^ Indications and contraindications for the use of NIPPV may not apply when the sole purpose of applying NIPPV is preoxygenation for intubation as the intubating clinician is physically present and prepared to intervene during this short period of time. When hypoxemic patients preoxygenated with NIPPV are removed from positive pressure for the intubation procedure, there is a risk of derecruitment of alveoli causing rapid desaturation. Maintaining continuous positive pressure during the intubation with the use of a nasal mask has been shown in the operating room to be beneficial in patients with hypoxemic respiratory failure and may be useful in the ED.^34^

Occasionally NIPPV is inadequate due to anatomic characteristics that make obtaining or maintaining an adequate mask seal difficult. In these patients that have significant mask leaks, or when higher pressures are required for preoxygenation, such as in patients with pulmonary edema or morbid obesity, supraglottic airways may be an option for preoxygenation. Current data for the use of supraglottic airways for preoxygenation in the ED are limited to a case report; however, there are some limited data showing success with prolonging safe apnea time in morbidly obese patients in the operating room.^35^,^36^ Limiting insufflation pressures to a maximum of 20cmH20 should not result in increased gastric distention or aspiration events.^28^,^37^,^38^

Pharmacologic assistance to decrease anxiety or even induce sedation may be useful if chosen carefully to improve patient tolerance of either the NIPPV mask or supraglottic airway. A recent observational study formalized a protocol, termed delayed sequence intubation (DSI), in which ketamine was administered to induce a dissociated state to allow preoxygenation with a NRB mask or NIPPV prior to the administration of a neuromuscular blocking agent, and showed most patients had improved preoxygenation.^39^

Apneic oxygenation, also known as “diffusion respiration” or “aventilatory mass flow,” occurs because oxygen removal from the aveoli by circulating blood brings alveolar pressure to slightly subatmospheric levels, generating a negative pressure gradient, drawing oxygen from the upper airway into the lungs.^40^ Nasal oxygen administration as a method of apneic oxygenation has repeatedly been shown to prolong safe apnea time, including in obese patients.^41^–^43^ Apneic oxygen supplementation has been found to prevent desaturation for as long as 100 minutes at the expense of severe hypercapnea and decreased pH in operative patients. However, the effects of hypercapnea during apnea can be deleterious leading to ventricular arrhythmias, neurologic compromise, and even death.^44^–^48^ Transnasal humidifed rapid-insufflation ventilatory exchange (THRIVE) has recently been shown to not only increase apnea time by delivering high-flow humidified oxygen via nasal cannula at 70L/pm, but also reduce the rate of carbon dioxide increase by gaseous mixing and flushing of dead space.^49^

Recent evidence with the use of a high-flow nasal cannula (HFNC) capable of delivering a humidified, adjustable FiO_2_ up to 60Lpm for preoxygenation and apneic oxygenation is mixed. Vourc’h and colleagues found no difference in desaturation rates when HFNC for preoxygenation and apneic oxygenation was compared to high-flow facemask in hypoxemic patients.^50^ However, Miguel-Montanes et al. found that preoxygenation and apneic oxygenation with the same HFNC reduced desaturation compared to NRB in patients intubated in the intensive care unit. HFNC resulted in higher O_2_ saturation after preoxygenation, during intubation, and at 5- and 30-minutes post-intubation.^51^ A benefit of HFNCs is varying amounts of continuous positive airway pressure achieved at higher flow rates.^52^ A low-cost, low-risk application of apneic oxygenation is via standard or wide-bore nasal prongs at 10–15L/pm. This flow rate is well tolerated,^53^ provides near 100% FiO_2_ to the nasopharynx during the apneic period and may prevent desaturation in some patients. For a more detailed description of preoxygenation and apneic oxygenation, see Weingart and Levitan’s comprehensive review.^54^

#### Recommendations

1. Preoxygenation and apneic oxygenation should be performed in all critically ill patients. Despite mixed data, apneic oxygenation is a low-risk intervention that may provide significant benefit in prolonging the safe apneic period. If a HFNC system is not available, a wide-bore nasal cannula or standard nasal prongs should be used to augment preoxygenation and provide apneic oxygenation.
2. In patients with shunt physiology due to atelectasis or alveolar filling from pneumonia, ARDS or pulmonary edema, NIPPV can improve alveolar recruitment and oxygenation. In select patients, supraglottic airways may be considered when higher pressures are needed or a mask seal with NIPPV cannot be achieved. One must balance this potential benefit of a supraglottic airway with the risk of aspiration or upper airway injury. Nasal continuous positive airway pressure with a nasal mask may be useful to maintain alveolar recruitment during intubation in patients at high risk.
3. For patients who cannot tolerate the NIPPV mask (e.g. delirium), analgesia, anxiolysis, or DSI may be considered to optimize preoxygenation. If procedural sedation for preoxygenation is performed, one must be prepared to intubate at the onset of DSI, even with ketamine, due risk of cardiac arrest, laryngospasm and apnea, which have all been reported with ketamine.^55^,^56^

### Hypotension

Peri-intubation hypotension is common and roughly one-quarter of patients develop transient hypotension after emergent intubation and transition to positive pressure ventilation.^57^ A recent report shows that nearly 30% of critically ill patients had cardiovascular collapse after intubation.^11^ Peri-intubation hypotension is a major risk factor for adverse events, including cardiopulmonary arrest related to airway management, longer intensive care unit stays and increased hospital mortality.^10^,^58^–^62^ Griesdale and colleagues report that a SBP<70mmHg complicates 10% of intubations in critically ill patients^6^ and pre-induction shock index (heart rate/systolic blood pressure) >0.8 and hypotension have been shown to predict patients at risk for post-intubation hypotension.^58^,^59^,^62^

Venous return to the heart is driven by the difference between venous pressure (i.e. mean systemic pressure) and right atrial pressure. During spontaneous respiration, the negative intrathoracic pressure augments this pressure gradient, which in essence “pulls” blood back to the right heart. Any physiologic disturbance that disrupts this driving pressure gradient will decrease venous return. Transition to positive pressure ventilation increases intrathoracic pressure and thus right atrial pressure, decreasing the pressure differential driving venous return.^63^–^66^ Common causes of shock such as volume depletion, capillary leak, or a loss of systemic vascular resistance will decrease the mean systemic pressure and venous return making these patients particularly susceptible to positive pressure ventilation induced hypotension.

Fluid resuscitation is important in critically ill patients, as an increase in circulating volume will increase mean systemic pressure and venous return.^65^,^67^ If the right heart can accommodate the increased venous return, the patient will be a “volume responder” and cardiac output will increase. Volume responsiveness is typically defined as an increase in cardiac output by >15% in response to a fluid challenge. Rapid evaluation of volume responsiveness is easily performed at the bedside by a number of techniques evaluating cardiopulmonary interactions, such as respiratory changes in inferior vena cava diameter, arterial waveform analysis, or Doppler assessment of aortic flow velocities.^68^ Not all patients will be volume responsive, in which case vasopressors may be helpful for maintaining vascular tone and perfusion pressure and norepinephrine is preferred vasopressor in critically ill patients.^69^,^70^ Pure vasoconstrictors such as phenylephrine will increase vascular resistance and blood pressure, but will depress the cardiac output and decrease venous return. In patients who are in shock, or under-resuscitated, this decrease in venous return and depressed cardiac output may actually worsen hemodynamics despite improved blood pressure.^64^ In patients with transient hypotension during intubation from vasodilation or a positive pressure induced decrease in venous return, peripherally administered vasopressors may be useful for maintaining adequate end-organ perfusion pressure until adequate fluid resuscitation is achieved. Diluted phenylephrine boluses may be useful for ameliorating the decrease in vascular tone induced by anesthetic agents and maintain systemic vascular resistance and diastolic perfusion of the coronary arteries until the transient hypotension resolves or fluid resuscitation can be optimized.^79^–^82^ When given for a short duration, peripherally administered vasopressors have been shown to be low risk.^71^

The choice of induction agents can contribute to peri-intubation hypotension as many have adverse hemodynamic effects. Benzodiazepines and propofol have a sympatholytic effect, leading to myocardial depression and a decrease in vascular tone.^72^ Etomidate is a non-benzodiazepine sedative, which has been shown to be relatively hemodynamically neutral.^73^,^74^ Ketamine is also an attractive choice for an induction agent given its sympathomimetic properties,^75^ although there have been reports of cardiac arrest after ketamine administration.^55^ Jabre and colleagues compared etomidate and ketamine for emergency intubation in septic patients and found no difference in serious complications.^76^ Although generally considered hemodynamically neutral, some neuromuscular blocking agents have indirect cardiovascular effects through histamine release and parasympathetic activity.^77^,^78^ Thus, pre-intubation fluid resuscitation and thoughtful pharmacologic intervention will optimize the hemodynamic stability with airway management in the hypotensive patient.

#### Recommendations

1. Patients with conditions that reduce venous return are particularly susceptible to hypotension and patients at risk are suggested by pre-intubation hypotension or an elevated shock index >0.8. These patients should be hemodynamically optimized prior to intubation. This includes aggressive volume resuscitation if the patient is likely to be a volume responder. Hemodynamically stable induction agents should be used when possible.
2. For patients unresponsive to volume resuscitation, a norepinephrine infusion should be initiated.
3. If pre-intubation resuscitation is not feasible due to impending cardiopulmonary arrest in patients with shock, peripherally administered vasopressor boluses can be prepared quickly at the bedside and may maintain blood pressure during intubation and resuscitation. This intervention has not been studied in critically ill adults; however, diluted epinephrine (given as 10–50mcg boluses with a concentration of 1–10mcg/mL) may be preferred due to its inotropic effect.
4. For patients without shock who have a transient drop in blood pressure after intubation due to the vasodilatory effects of induction agents or transition to positive pressure ventilation, diluted phenylephrine (given as 50–200mcg boluses with a concentration of 100mcg/mL) may be useful.

### Severe metabolic acidosis

When acidemia develops from a respiratory acidosis, rapid correction of that acidemia can occur by increasing the alveolar ventilation. Doubling the alveolar ventilation will reduce the PaCO_2_, roughly by half. Respiratory acidosis is then usually corrected easily by interventions that increase the alveolar ventilation such as bag-valve mask ventilation, NIPPV, or mechanical ventilation. When acidemia develops from a metabolic acidosis, maintenance of acid-base homeostasis depends on a compensatory respiratory alkalosis from alveolar hyperventilation.^83^ Unlike the rapid decrease in PaCO_2_ possible during hypoventilatory states, when hypocapnia is already present due to a compensatory respiratory alkalsosis, further hyperventilation results in incrementally smaller decreases in PaCO_2_ and eventually reaches a plateau at which point there is no effect of further increasing alveolar ventilation.^83^ Thus, in severe metabolic acidosis from diseases such as diabetic ketoacidosis (DKA), salicylate toxicity, and even severe lactic acidosis, the organic acid production demands an alveolar ventilation requirement that sometimes cannot be met and patients can subsequently develop profound acidemia. In the event that patients with severe acidemia require intubation, even a brief apneic period can lead to a precipitous drop in pH given the loss of the already inadequate respiratory compensation. Further, the pre-intubation alveolar ventilation sometimes cannot be matched by the mechanical ventilator, which has physical limits on the volume and rate that can be delivered. For example, a patient with DKA and Kussmaul respirations may have a minute ventilation of >40L due to a respiratory rate of 40 breaths per minute and a tidal volume of >1L. Mechanically ventilating this patient with a set rate of 30 and tidal volume of 1L will result in an inadequate minute ventilation of 30L. Consequently, even if lung protective ventilation strategies are abandoned, the maximal attainable minute ventilation may be less than the pre-intubation minute ventilation, leading to a precipitous drop in pH and a high risk of hemodynamic deterioration after intubation. Patients with extremely high minute ventilation requirements are at high risk of developing relative hypoventilation, flow starvation, patient-ventilator dysynchrony and worsened acidosis. In these situations, a pressure-targeted mode, such as pressure support ventilation or pressure control, may allow better patient-ventilator synchrony and maintenance of the minute ventilation, especially in the spontaneously breathing patient.

#### Recommendations

1. Intubation should be avoided, if possible, in patients with severe metabolic acidosis who have a minute ventilation requirement not likely to be met by the mechanical ventilator, despite a low pH. A short trial of NIPPV may adequately support the respiratory work of breathing until correction of the underlying metabolic acidosis can occur and will provide an estimate of the patient’s intrinsic minute ventilation by measuring the patient’s respiratory rate and tidal volume delivered with each breath.
2. If intubation is necessary, maintaining spontaneous respiration becomes the critical action both during intubation and with mechanical ventilation. This will allow the patient to maintain their own high minute ventilation and includes using sedative agents that are less likely to reduce the patient’s respiratory drive. Rapid sequence intubation should be avoided if possible, and if one is deemed necessary, a short-acting neuromuscular blocker such as succinylcholine should be used.
3. After intubation, we recommend choosing a ventilator mode that allows the patient to set and maintain their own minute ventilation in order to best maintain their respiratory compensation. A pressure-targeted ventilator mode such as pressure support ventilation or pressure control mode will allow the patient to set the rate and tidal volume received. Special care should be taken to monitor for air trapping given the high rates and tidal volumes reached as well as monitor for respiratory muscle fatigue, which will result in a loss of compensation.

### Right Ventricular Failure

Under normal circumstances, the right ventricle is a low-pressure, high-compliance, flow-based chamber geared to propel venous blood returning to the heart into the pulmonary circulation.^84^,^85^ However, any process that increases right ventricular (RV) afterload, such as chronic pulmonary hypertension from lung or left ventricular disease, pulmonary arterial hypertension, or acute pulmonary embolism strains the RV, which adapts by increasing both contractility and preload.^86^,^87^ The critical action for the emergency physician is to determine if the patient has RV dysfunction, where the RV has some reserve and is able to perform some of its pumping function, or overt RV failure, in which the RV is unable to meet increased demands leading to RV dilation, retrograde flow, decreased coronary perfusion, and ultimately systemic hypotension and cardiovascular collapse.^85^

Intrathoracic pressure changes with respiration have an exaggerated effect on hemodynamics in the patient with RV failure, worsening cardiopulmonary interactions and making intubation extremely risky. Unlike left ventricular function, which improves with positive pressure ventilation, RV function worsens with the increase in intrathoracic pressure induced by positive pressure ventilation. This occurs because the intrathoracic pressure is transmitted to the alveolar capillary bed, leading to collapse of these small vessels and increases the pulmonary vascular resistance against which the RV must pump.^88^ When patients with RV failure require intubation, the increased RV afterload and decreased preload associated with invasive mechanical ventilation can often lead to cardiovascular collapse.^89^ When possible, work of breathing and gas exchange should be supported with medications, oxygen, and if positive pressure ventilation is needed then NIPPV and low positive end-expiratory pressure with the goals of decreasing work of breathing, limiting atelectasis, and reducing hypoxic vasoconstriction. These methods of support allow the patient to breathe spontaneously, resulting in a smaller rise in intrathoracic pressure than control modes.

Patients with increased RV afterload often present with varying degrees of RV strain on bedside echocardiography, including a dilated RV and inferior vena cava, septal flattening during systole in pressure overloaded states, and septal flattening during diastole in volume overloaded states. While patients with RV dysfunction may respond to small fluid challenges or an inotropic agent, further increasing preload with a fluid challenge in patients with RV failure is unlikely to be fruitful, and may be deleterious as volume overloading a pressure overloaded RV increases diastolic wall tension and left ventricular diastolic dysfunction, directly worsening left ventricular filling and stroke volume.^87^ Thus, determining volume responsiveness is quite challenging and critically important as the volume-starved left ventricle will always appear volume responsive when using the usual techniques such as pulse pressure variation (PPV) or stroke volume variation (SVV). The tricuspid valve regurgitation jet velocity, tricuspid annular plane systolic excursion (TAPSE), tricuspid annular peak velocity or isovolumetric contraction velocity (IVV) and RV outflow tract velocity-time integral are easy to perform and useful methods of determining the degree of RV strain, volume responsiveness, and contractile reserve on bedside echocardiography.^90^,^91^ Hemodynamic optimization, including RV afterload reduction with inhaled pulmonary artery vasodilators such as inhaled nitric oxide (iNO) or inhaled epoprostenol (Flolan), should be performed in patients with RV failure prior to intubation to avoid cardiovascular collapse with positive pressure ventilation. For a more detailed review of hemodynamic assessment methods, see Dalabih et al. and Krishnan et al.^90^,^91^

#### Recommendations

1. Bedside echocardiographic assessment of RV function should be performed to assess RV dysfunction versus RV failure. If the patient has some contractile reserve (RV dysfunction), cautious fluid resuscitation should be performed.
2. Preoxygenation is essential despite the difficulties resulting from intracardiac shunt and ventilation-perfusion (V/Q) mismatch, which commonly occur in right heart failure.^28^
3. Apneic oxygenation should be performed given the potential for benefit.^54^ INO at low concentrations (<30ppm), delivered in-line continuously through the nasal cannula, can augment oxygenation by improving V/Q matching in the hypoxemic patient but may worsen V/Q mismatch at higher concentrations. In the RV failure patient without hypoxemia, 30–80ppm of iNO delivered through the nasal cannula, or inhaled epoprostenol during preoxygenation and apneic oxygenation can reduce pulmonary vascular resistance.^90^
4. Induction agents should be considered carefully. Hemodynamically neutral sedatives such as etomidate should be used for induction. Intravenous fentanyl premedication may be useful to blunt the hypertensive response to laryngoscopy.
5. Continuous norepinephrine infusion should be started prior to induction in hypotensive patients with the goal of increasing mean arterial pressure higher than pulmonary artery pressure, which can be determined by bedside echocardiography. For patients without hypotension, norepinephrine should be primed and “in-line” in the event of post intubation or sedative induced hypotension.
6. The goals of mechanical ventilation include maintenance of a low mean airway pressure and avoidance of hypoxemia, atelectasis, and hypercapnea, which increase RV afterload.^92^–^95^

## CONCLUSION

The difficult airway is well recognized as a clinical entity and is classically based on anatomic considerations. In this paper we describe another aspect of the difficult airway that involves physiologic abnormalities that must be considered in developing an intubation plan. These physiologic abnormalities must be considered and addressed prior to intubation. If they are not, significant untoward outcomes can result. We present four physiologic disturbances that must be considered carefully when planning for and performing tracheal intubation in the ED to avoid complications from the very procedure intended to be life saving. Many of the recommendations presented are based on clinical experience and physiologic principles and thus represent opportunities for formal investigation.
